# Analysis of Male Sex as a Risk Factor in Older Adults With Coronavirus Disease 2019: A Retrospective Cohort Study From the New York City Metropolitan Region

**DOI:** 10.7759/cureus.9912

**Published:** 2020-08-21

**Authors:** Ashutossh Naaraayan, Abhishek Nimkar, Amrah Hasan, Sushil Pant, Momcilo Durdevic, Henrik Elenius, Corina Nava Suarez, Stephen Jesmajian

**Affiliations:** 1 Internal Medicine, Montefiore New Rochelle Hospital, Albert Einstein College of Medicine, New Rochelle, USA

**Keywords:** coronavirus disease 2019 (covid-19), sex differences, geriatric vs. non-geriatric, age and ageing, outcome analysis, outcome disparities, covid-19 pneumonia, oxygen support, acute kidney injury, acute liver injury

## Abstract

Background

Advancing age and male sex have been identified as risk factors for poor outcomes in coronavirus disease 2019 (COVID-19). However, there is a dearth of data investigating the impact of age on the risk reported with male sex. We aimed to determine the risk associated with male sex in people of different age groups, that is, in people younger or older than 65 years of age.

Methods

This is a retrospective cohort study that included 370 adult patients hospitalized with COVID-19 between March 12, 2020, and May 13, 2020, at a 242-bed teaching community hospital in the New York City metropolitan region. Patients were classified into younger (age<65 years, n=132) and older individuals (age>=65, n=238). We calculated odds ratios for poor outcomes in men compared to women separately in these two groups.

Results

Among older individuals, there was no difference in the odds of poor outcomes between men and women. In contrast, among younger people, men had higher odds of severe pneumonia, need for high oxygen support, acute kidney injury and acute liver injury when compared to women.

Conclusions

Among people older than 65 years, sex did not impact disease severity and outcomes in COVID-19. Thus, older women were equally likely to have severe COVID-19 when compared to age-matched men. In contrast, among younger middle-aged adults (29-64 years), men had higher odds of end-organ damage from COVID-19 compared to women. Based on these observations, age is a more important driver of poor outcomes in COVID-19 than sex. Public health policies need to create awareness for the increased risk of older individuals to COVID-19, regardless of sex.

## Introduction

At the time of writing, 13 million cases of coronavirus disease 2019 (COVID-19) caused by the severe acute respiratory syndrome coronavirus-2 (SARS-CoV-2) have been described worldwide, resulting in 571,000 deaths [1]. Laboratory-confirmed cases have been documented from 188 countries on six continents, with Europe and the US reporting the highest number of cases and deaths. Through March and April 2020, the New York metropolitan region was the epicenter for COVID-19 [2]. Even though the peak of the infection has passed in Europe and China, a concerning number of cases and deaths are being reported from the US, India, Brazil and other nations [2].

Advancing age and male sex have been independently identified as risk factors for poor outcomes in COVID-19 illness [3-6]. However, there is a dearth of data describing the impact of sex on outcomes in older individuals (age>=65 years). Here, we present data analyzing outcomes in COVID-19 hospitalizations, from one of the earliest epicenters in the US [7]. We specifically investigated the risk associated with male sex in people of different age-groups, that is, in people younger or older than 65 years of age.

## Materials and methods

Study design and participants

This retrospective case series included adult (>18 years old) patients consecutively hospitalized with confirmed COVID-19 illness between March 12, 2020, and May 13, 2020, at a 242-bed teaching community hospital in the New York City metropolitan area. The hospital serves approximately 250,000 people in the southern Westchester County, New York. Cases were confirmed through positive results for the SARS-CoV-2 virus by reverse-transcriptase polymerase chain reaction (PCR) testing of a nasopharyngeal swab specimen.

Outcome data was followed up until June 15, 2020. Of the 377 patients admitted during the study period, those who were transferred to another facility for tertiary level care (n=5) and those who left against medical advice (n=2) were excluded from the analysis. After said exclusions, final analysis included 370 patients.

Data collection

Data was manually abstracted from electronic health records by the authors, and included demographics, comorbid conditions, laboratory data and outcomes. Three authors (AN, AN and SP) independently reviewed the data for accuracy.

Definitions of comorbidities

Cardiac disease was defined as chronic heart conditions including but not limited to coronary artery disease, previous myocardial infarction, cardiac arrhythmias and congestive heart failure. Renal disease was defined as the presence of chronic kidney disease with or without the need for dialysis. Malignancy was defined as the presence of an active malignancy or a history of a previous malignancy at any time in the patient's life. Body mass index (person's weight in kilograms divided by the square of height in meters) was calculated to account for obesity (body mass index >30 kg/m^2^). Patients were classified into one of the four categories for race/ethnicity: Whites, Blacks, Hispanic ethnicity of any race and Others.

Definition of outcomes

Severe COVID-19 manifestations were considered as outcomes and included the following: (1) severe pneumonia, defined as pneumonia with a radiological evidence of bilateral infiltrates with a respiratory rate >30 breaths/minute or oxygen saturation <90% on room air, per the World Health Organization (WHO) guidelines [8]; (2) severe acute respiratory distress syndrome (ARDS), defined as the ratio of arterial oxygen partial pressure to fractional inspired oxygen of <=100 on a positive end-expiratory pressure >=5 cm of water, as per the Berlin guidelines [9]; (3) high-oxygen (HiO_2_) support requirement, defined as hypoxemia needing supplementation of oxygen at a flow rate >=10 l/minute via any oxygen delivery method; (4) acute kidney injury (AKI), defined as per the criteria from the Kidney Disease: Improving Global Outcomes, and the International Society of Nephrology [10]; (5) acute cardiac injury (ACI), defined as blood levels of hypersensitive troponin I above the 99th percentile upper reference limit (>0.03 ng/ml); (6) acute liver injury (ALI), defined as an elevation of liver enzymes (aspartate transaminase or alanine transaminase) greater than three times the upper limit of normal range (>126 units/l, normal range 0-42 units/l); (7) death, defined as in-hospital mortality.

Clinical data was missing for certain outcomes that accounted for the different denominators (n) for the outcome data. As an example, in-hospital death was assessed after excluding patients who did not yet have a definite outcome of mortality or discharge, that is, patients who were still being treated at the time of writing (n=5). Thereby, the denominator (n) for outcome of death was 365 and not 370. For most outcomes, data was missing for less than 10% of observations except for acute cardiac injury, which had troponin results available in ~53% of observations. Missing data was accounted for during analysis.

Outcome measures and statistical analysis

The study was done according to STrengthening the Reporting of OBservational studies in Epidemiology (STROBE) guidelines for observational studies [11]. We computed median with inter-quartile range, frequency, and percentages as our descriptive variables. Differences in percentage and median were assessed using the chi-squared test and Mann-Whitney test, respectively.

To determine independent association between the patient’s sex and outcomes, univariable and multivariable logistic regression models were used to estimate odds ratios. For the multivariable models, the following clinical covariates were adjusted for: age, race/ethnicity, hypertension, diabetes, cardiac disease, chronic kidney disease, chronic obstructive pulmonary disease, malignancy and patient’s body mass index. These analyses were done separately among the younger (age<65 years) and the older individuals (age>=65 years). A separate subgroup analysis was done for individuals aged>=80 years. Stata version 16.0 (StataCorp, Houston, TX) was used for analysis. Two-sided p<.05 was considered statistically significant.

Statement of ethics

The study was carried out in accordance with the Declaration of Helsinki, and was approved by the departmental research review committee with a waiver of informed consent due to its retrospective design (approval number 20.6.02).

## Results

Among the 370 patients, median age was 71 (59-82) years; 55.9% were women and 33.8% were White. One hundred thirty-two people were classified as younger (age<65 years) while 238 people were classified as older (age>=65 years) individuals. Older adults were less likely to be men (50.4% vs 65.9%, p=.004), less likely to be Hispanic and more likely to be White when compared to younger adults (Table 1). Older individuals were more likely to have a higher need for HiO_2_, and a higher incidence of AKI, ACI and in-hospital death when compared to younger individuals (Table 1).

**Table 1:** Patient characteristics and outcomes in younger (<65 years) and older (>=65 years) individuals hospitalized with COVID-19 ARDS: acute respiratory distress syndrome; BMI: body mass index; COPD: chronic obstructive pulmonary disease; HiO_2_: high oxygen; IQR: inter-quartile range; COVID, coronavirus disease 2019 Data is presented as number (percentage), except for age and BMI.

Characteristic	All patients (n=370)	Younger individuals, age<65 (n=132)	Older individuals, age>=65 (n=238)	p-value
Demographics				
Age (years), median (IQR)	71 (59–82)	55.5 (47–60)	79 (72–86)	<.001
Male	207 (55.9)	87 (65.9)	120 (50.4)	.004
Race				
Whites	125 (33.8)	29 (21.9)	96 (40.3)	<.001
Blacks	130 (35.1)	41 (31.1)	89 (37.4)	.2
Hispanics	75 (20.3)	47 (35.6)	28 (11.8)	<.001
Others	40 (10.8)	15 (11.4)	25 (10.5)	.8
Comorbidities				
Hypertension	245 (66.2)	62 (46.9)	183 (76.9)	<.001
Diabetes	157 (42.4)	52 (39.4)	105 (44.1)	.4
Cardiac disease	121 (32.7)	20 (15.2)	101 (42.4)	<.001
Chronic kidney disease	41 (11.1)	8 (6.1)	33 (13.9)	.02
COPD	50 (13.5)	6 (4.6)	44 (18.5)	<.001
Malignancy	72 (19.5)	13 (9.9)	59 (24.8)	<.001
BMI (kg/m^2^), median (IQR)	27.4 (24–32.1)	29.4 (25.9–35.5)	26 (23.1–30)	<.001
Outcomes				
Severe pneumonia	239/370 (64.6)	81/132 (61.4)	158/238 (66.4)	.3
Severe ARDS	80/319 (25.1)	32/125 (25.6)	48/194 (24.7)	.9
HiO_2_ requirement	208/370 (56.2)	65/132 (49.2)	143/238 (60.1)	.04
Acute kidney injury	182/331 (54.9)	53/120 (44.2)	129/211 (61.1)	.003
Acute cardiac injury	112/197 (56.9)	20/64 (31.3)	92/133 (69.2)	<.001
Acute liver injury	74/368 (20.1)	23/131 (17.6)	51/237 (21.5)	.4
In-hospital death	150/365 (41.1)	27/128 (21.1)	123/237 (51.9)	<.001

Among younger people, men were more likely to have severe pneumonia, need for HiO_2_, AKI, and ALI when compared to women (Table 2). In contrast among older individuals, there was no significant sex difference between most outcomes (Table 2).

**Table 2:** Patient characteristics and outcomes in younger and older individuals analyzed by patient’s sex ARDS: acute respiratory distress syndrome; BMI: body mass index; COPD: chronic obstructive pulmonary disease; HiO_2_: high oxygen; IQR: inter-quartile range Data is presented as number (percentage), except for age and BMI.

Characteristic	Younger individuals (age<65 years)			Older individuals (age>=65 years)		
	Men (n=87)	Women (n=45)	p-value	Men (n=120)	Women (n=118)	p-value
Demographics						
Age (years), median (IQR)	55 (44–60)	56 (50–60)	.6	77 (71–85)	80 (74–88)	.02
Male	17 (19.5)	12 (26.7)	.4	48 (40)	48 (40.1)	.9
Race						
Whites	22 (25.3)	19 (42.2)	.046	44 (36.7)	45 (38.1)	.8
Blacks	40 (45.9)	7 (15.6)	<.001	14 (11.7)	14 (11.9)	.9
Hispanics	8 (9.2)	7 (15.6)	.3	14 (11.7)	11 (9.3)	.6
Comorbidities						
Hypertension	40 (45.9)	22 (48.9)	.8	93 (77.5)	90 (76.3)	.8
Diabetes	35 (40.2)	17 (37.8)	.8	58 (48.3)	47 (39.8)	.2
Cardiac disease	12 (13.8)	8 (17.8)	.6	53 (44.2)	48 (40.7)	.6
Chronic kidney disease	4 (4.6)	4 (8.9)	.3	16 (13.3)	17 (14.4)	.8
COPD	4 (4.6)	2 (4.4)	.9	25 (20.8)	19 (16.1)	.4
Malignancy	5 (5.8)	8 (17.8)	.03	33 (27.5)	26 (22.03)	.3
BMI (kg/m^2^), median (IQR)	29 (26–36)	29 (24–35)	.8	26 (23–29)	27 (23–31)	.3
Outcomes						
Severe pneumonia	60/87 (68.9)	21/45 (46.7)	.01	78/120 (65)	80/118 (67.8)	.7
Severe ARDS	26/84 (30.9)	6/41 (14.6)	.05	18/99 (18.2)	30/95 (31.6)	.03
HiO_2_ requirement	49/87 (56.3)	16/45 (35.6)	.02	70/120 (58.3)	73/118 (61.9)	.6
Acute kidney injury	40/78 (51.3)	13/42 (30.9)	.03	64/108 (59.3)	65/103 (63.1)	.6
Acute cardiac injury	16/47 (34)	4/17 (23.5)	.4	46/66 (69.7)	46/67 (68.7)	.9
Acute liver injury	20/86 (23.3)	3/45 (6.7)	.02	27/119 (22.7)	24/118 (20.3)	.7
In-hospital death	20/83 (24.1)	7/45 (15.6)	.3	60/119 (50.4)	63/118 (53.4)	.7

**Figure 1:**
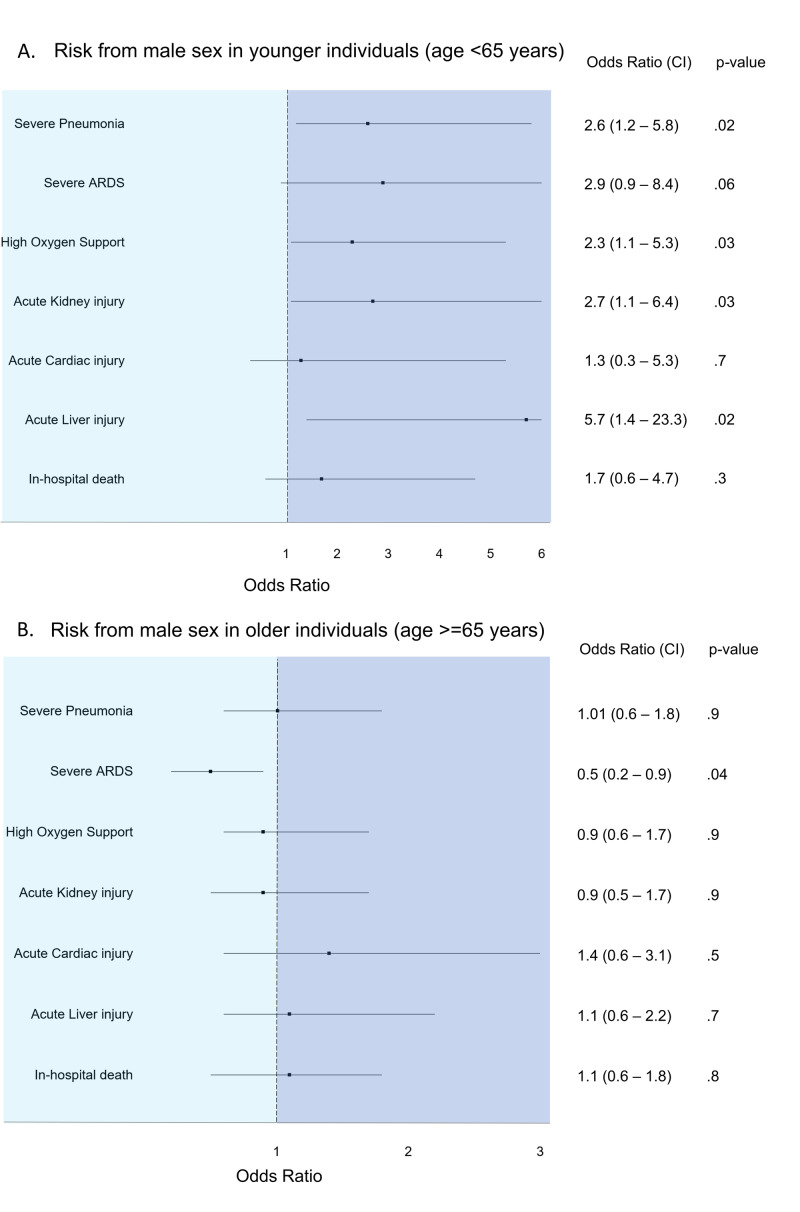
Risk from male sex in (A) younger (age<65 years) and (B) older (age>=65 years) individuals ARDS: acute respiratory distress syndrome

Among younger individuals, men had significantly higher odds of severe pneumonia, need for HiO_2_, AKI and ALI, when compared to women (Table 3, Figures 1A, 1B).

**Table 3:** Risk for poor outcomes for men compared to women among younger (age<65 years) and older individuals (age>=65 years) ARDS: acute respiratory distress syndrome; CI: confidence interval; HiO_2_: high oxygen p-values for the multivariable models were derived after adjusting for age, race/ethnicity, hypertension, diabetes, cardiac disease, chronic kidney disease, chronic obstructive pulmonary disease, malignancy and patient’s body mass index.

Odds of outcomes in hospitalized men compared to women				
	Univariable analysis, odds ratio (CI)	p-value	Multivariable analysis, odds ratio (CI)	p-value
Outcomes in younger individuals (age<65 years)				
Severe pneumonia	2.5 (1.2–5.3)	.01	2.6 (1.2–5.8)	.02
Severe ARDS	2.6 (0.9–6.9)	.06	2.9 (0.9–8.4)	.06
HiO_2_ requirement	2.3 (1.1–4.9)	.03	2.3 (1.1–5.3)	.03
Acute kidney injury	2.4 (1.1–5.2)	.03	2.7 (1.1–6.4)	.03
Acute cardiac injury	1.7 (0.5–5.9)	.4	1.3 (0.3–5.3)	.7
Acute liver injury	4.2 (1.2–15.2)	.03	5.7 (1.4–23.3)	.02
In-hospital death	1.6 (0.6–4.2)	.3	1.7 (0.6–4.7)	.3
Outcomes in older individuals (age>=65 years)				
Severe pneumonia	0.9 (0.5–1.5)	.7	1.01 (0.6–1.8)	.9
Severe ARDS	0.5 (0.3–0.9)	.03	0.5 (0.2–0.9)	.04
HiO_2_ requirement	0.9 (0.5–1.5)	.6	0.9 (0.6–1.7)	.9
Acute kidney injury	0.9 (0.5–1.5)	.6	0.9 (0.5–1.7)	.9
Acute cardiac injury	1.1 (0.5–2.2)	.9	1.4 (0.6–3.1)	.5
Acute liver injury	1.2 (0.6–2.1)	.7	1.1 (0.6–2.2)	.7
In-hospital death	0.9 (0.5–1.5)	.6	1.1 (0.6–1.8)	.8

Among older individuals, however, there was no significant difference between the odds for most outcomes between men and women. This lack of difference between men and women was observed in an even older subgroup (age>=80 years) (Table 4).

**Table 4:** Risk for poor outcomes for men compared to women among patients aged 80 years or older ARDS: acute respiratory distress syndrome; CI: confidence interval; HiO_2_: high oxygen. p-values for the multivariable models were derived after adjusting for age, race/ethnicity, hypertension, diabetes, cardiac disease, chronic kidney disease, chronic obstructive pulmonary disease, malignancy and patient’s body mass index.

Outcomes	Univariable analysis, odds ratio (CI)	p-value	Multivariable analysis, odds ratio (CI)	p-value
Severe pneumonia	0.9 (0.4–2.1)	.8	0.9 (0.3–2.2)	.7
Severe ARDS	0.4 (0.1–1.3)	.1	0.3 (0.1–1.1)	.07
HiO_2_ requirement	0.9 (0.4–2.1)	.9	0.9 (0.4–2.1)	.8
Acute kidney injury	1.1 (0.5–2.6)	.8	1.2 (0.5–3.1)	.7
Acute cardiac injury	0.9 (0.3–3.4)	.9	0.9 (0.3–3.8)	.9
Acute liver injury	2.1 (0.9–4.9)	.09	2.1 (0.8–5.2)	.1
In-hospital death	0.9 (0.4–1.9)	.8	0.8 (0.4–1.9)	.7

## Discussion

Ours is one of the first studies on COVID-19 patients that investigated the effect of a person’s sex on outcomes in different age groups. We found a significantly higher risk of severe illness manifested by end-organ damage among younger men compared to women of comparable age (29-64 years). However, among older adults (age>=65 years), male sex was not associated with a more severe illness and worse outcomes.

In the previous influenza and novel coronavirus epidemics, SARS-CoV-1 and Middle East respiratory syndrome (MERS), in addition to virus-induced cytopathic effects, an excessive and dysregulated host immune response played a crucial role in the pathology and mortality [12,13]. This exaggerated response with supraphysiologic levels of cytokines is referred to as a “cytokine storm”. During viral epidemics, sex difference in outcomes thus depend on the balance between the beneficial anti-viral immune response and the harm from an exaggerated inflammatory response.

Historically, influenza epidemics have varied in their risk to the sexes: some were worse for men (1918) and others for women (2004) [14]. However, men have consistently had worse outcomes and higher mortality rates in the coronavirus outbreaks (SARS and MERS) [13]. Likewise, COVID-19 hospitalizations have mostly comprised men, and men have been identified to be at greater risk by many researchers [4-7,15]. However, only two studies to date have investigated the impact of sex on outcomes in patients stratified by age [6,15]. Both studies described the increased risk of death with male sex regardless of age. However, the studies had major limitations: a third of patients still hospitalized in one at the time of reporting and concern for overlap of patients among the different studies in the meta-analysis.

Similar to our study, a recent report by the Centers for Disease Control and Prevention (CDC), based on a large national database of 1.3 million COVID-19 cases from the US, indeed shows the varying effect of sex with age in COVID-19 [16]. Men had a higher incidence of COVID-19 in the middle-age groups (30-79 years) while women had a higher incidence of COVID-19 in adults aged <30 years and those aged >=80 years. In another report that included 2.9 million COVID-19 cases from 116 countries, the United Nations (UN) Women and WHO share similar findings of an increased prevalence of COVID-19 in men in the middle-age groups and a dissipation of the risk from male sex with advancing age [17]. Our findings of increased risk among men in the middle-age group (29-64 years in our study) are certainly consistent with the CDC and the UN report. Additionally, in our study, the lack of difference in outcomes between the two sexes persisted in adults >=80 years of age. We could not test the sex difference in outcomes in patients younger than 30 years of age as in our study only one hospitalized patient was under the age of 30.

There are several mechanisms by which men could be at a higher risk from COVID-19 than women. Sex hormones and activity of X-linked genes, both of which modulate the innate and adaptive immune response to viral infection, might be involved [18]. Channappanavar et al. demonstrated that male mice infected with SARS-CoV-1 were more susceptible to the infection compared to age-matched females [13]. Furthermore, the degree of sex bias to SARS-CoV-1 infection in middle-aged mice was more pronounced compared to young mice. These findings are consistent with the CDC and the UN report, as well as our own experience in the current study. In addition, the authors reported an enhanced susceptibility of ovariectomized and estrogen receptor antagonist-treated female mice, demonstrating the protective effect of estrogen receptor signaling in females. Because the X chromosome contains a high density of immune-related genes, women generally mount stronger innate and adaptive immune responses than men [19]. This results in faster clearance of pathogens and greater vaccine efficacy in women than in men.

Another biological difference may relate to sex differences in angiotensin-converting enzyme 2 (ACE2) receptors. The primary route of SARS-CoV-2 infection is via the ACE2 receptors. Men have been shown to have higher circulating levels of ACE2 and higher levels of ACE2 in the lungs compared to women [15,20]. In addition, there are marked differences in the density of ACE2 receptors in the reproductive organs: the testes have much higher levels of ACE2 than the ovaries [21]. Thus, the higher susceptibility to COVID-19 in men may be from a direct cytopathic effect from the virus by increased ACE2-mediated cellular entry. Indeed, in SARS-CoV-1 infection, which also utilizes ACE2 as its receptor, men were shown to have higher viral loads compared to women [22]. Shastri et al. demonstrated that men had significantly delayed SARS-CoV-2 viral clearance when compared to women (median difference of two days in achieving a negative PCR result, p=.038) [23]. The potential role of testes as a reservoir of infection was suggested by the authors [23].

In addition, an abnormal and dysregulated cytokine response is likely partly responsible for the sex disparity in disease outcomes. In COVID-19, men demonstrated lower expression of cytokines with protective effects against viral infection, including CCL2, CCL3, CCL4 and CXCL16, than women [15]. In contrast, pro-inflammatory cytokines and chemokines, including CCL14, CCL23, IL7, IL16 and IL18, were preferentially expressed in men, underlying the higher susceptibility of men developing cytokine release syndrome in COVID-19.

In addition to the biologic reasons described above, the disproportionate risk in men may partly be explained by higher risk behaviors such as smoking and alcohol use, and occupational exposure [17]. There may be other behavioral differences that favor women, with prior studies suggesting women are more likely than men to follow hand hygiene practices and seek preventive care [24,25].

Our findings beg the question, why is the increased risk from male sex not observed in older adults with COVID-19? Observations of enhanced mortality in all elderly, regardless of sex, have been reported consistently from all regions of the world, China, Europe and America [4-7]. Because death is a terminal event, the high mortality rate might overpower and mask any significant increased risk from the male sex in older individuals. Interestingly, age was consistently reported as a risk factor during the previous coronavirus epidemics, SARS and MERS, as well [13]. The current study confirmed that increasing age was associated with poor prognosis in these patients. Increased mortality with advancing age is at least in part due to a dysregulated immune response [26].

Studies in macaques revealed that in response to a viral infection, older macaques had a stronger innate immune response and a pro-inflammatory type-2 cytokine response, while manifesting a weaker adaptive immune response (T-cell and B-cell dysfunction) and decreased type-1 cytokines [27]. Similarly, in humans, a tendency for a stronger inflammatory response, potentially leading to cytokine-storm-related injury in the lungs of older individuals was observed [28]. These maladaptive immune responses with advancing age have previously been identified as a major cause of high mortality due to severe pneumonia in older adults. In addition to the inflammatory hyper-response, the humoral and cellular immune functions decline with age: interferon and immunoglobulin M levels decrease, the number of T cells decrease, cell division and proliferation decreases, and neutrophil chemotaxis and phagocytosis also decreases [29]. With fewer T cells, the ability to mount effective anti-viral response is diminished in older individuals. In addition to a lack of antiviral activity, a decline in host immunity after viral infections may lead to secondary bacterial infections. Overall, these changes with older age could lead to a defective innate and adaptive immune response, a deficiency in control of viral replication, and a more prolonged proinflammatory response, potentially leading to poor outcomes in this age group.

Strengths and limitations

Our study has some limitations. Due to the retrospective study design, not all laboratory tests were done in all patients and thus there was some missing data. We accounted for the missing data in our statistical analysis to resolve this issue. The study population was from a single hospital. In addition, because of the make-up of our cohort, we could not assess the impact of sex on outcomes among people under the age of 30 years. Strengths of the study include the accuracy of data that was manually extracted from patient charts and a relatively large study cohort. Additionally, the consistency of our findings in univariable and multivariable models points to the robustness of our results.

## Conclusions

In summary, the evidence tells us that among people older than 65 years, sex did not impact disease severity and outcomes in COVID-19. Thus, older women were equally likely to have severe illness manifesting as end-organ damage when compared to age-matched men. In contrast, among middle-aged adults, men had higher odds of severe illness compared to women. This age-dependent impact of the patient’s sex on outcomes in COVID-19 is a novel finding, and of great public health importance. Our findings are consistent with recent reports from the CDC and the UN that describe an increased incidence and prevalence of COVID-19 in men only in the middle-age group. Based on these observations, we conclude that age is a more important driver of poor outcomes in COVID-19 than sex.

The COVID-19 pandemic is a global public health emergency that has changed the world in an unprecedented way. Confirmation of our observation of an equitable risk in older men and women with further data will help guide recommendations for prevention, hospitalization and management of these individuals. Further collecting and analyzing sex and age-disaggregated data to clarify risks is thus essential. For now, informing the general public of a lower risk for severe illness in older women compared to age-matched men based on the previous literature, most of which did not specifically analyze the impact of sex in older people, would be inaccurate and falsely reassuring. Public health policies need to create awareness for the vulnerability of older individuals to COVID-19 regardless of sex. Our findings are of great public health importance and should be shared, corroborated and elucidated.

## References

[1] (2020). COVID-19 dashboard by the Center for Systems Science and Engineering (CSSE) at Johns Hopkins University & Medicine. https://coronavirus.jhu.edu/map.html.

[2] (2020). New York City region is now an epicenter of the coronavirus pandemic. https://www.nytimes.com/2020/03/22/nyregion/Coronavirus-new-York-epicenter.html.

[3] Zhou F, Yu T, Du R (2020). Clinical course and risk factors for mortality of adult inpatients with COVID-19 in Wuhan, China: a retrospective cohort study. Lancet.

[4] Williamson EJ, Walker AJ, Bhaskaran K (2020). Factors associated with COVID-19-related death using OpenSAFELY. Nature.

[5] Grasselli G, Zangrillo A, Zanella A (2020). Baseline characteristics and outcomes of 1591 patients infected with SARS-CoV-2 admitted to ICUs of the Lombardy region, Italy. JAMA.

[6] Docherty AB, Harrison EM, Green CA (2020). Features of 20 133 UK patients in hospital with covid-19 using the ISARIC WHO clinical characterisation protocol: prospective observational cohort study. BMJ.

[7] (2020). In an early US coronavirus hot spot, business slowly reopens. https://www.washingtonpost.com/business/cuomo-rings-opening-bell-as-nyse-reopens-after-2-months/2020/05/26/ff1d4c82-9f56-11ea-be06-af5514ee0385_story.html.

[8] (2020). Clinical management of COVID- 19: interim guidance. https://www.who.int/publications/i/item/clinical-management-of-covid-19.

[9] ARDS Definition Task Force (2012). Acute respiratory distress syndrome: the Berlin definition. JAMA.

[10] Kidney Disease: Improving Global Outcomes (KDIGO) Acute Kidney Injury Work Group (2012). KDIGO Clinical Practice Guideline for Acute Kidney Injury. Kidney Int Suppl.

[11] Von Elm E, Altman DG, Egger M, Pocock SJ, Gøtzsche PC, Vandenbroucke JP, for the STROBE Initiative (2008). The Strengthening the Reporting of Observational Studies in Epidemiology (STROBE) statement: guidelines for reporting observational studies. J Clin Epidemiol.

[12] Liu Q, Zhou Y-H, Yang Z-Q (2016). The cytokine storm of severe influenza and development of immunomodulatory therapy. Cell Mol Immunol.

[13] Channappanavar R, Fett C, Mack M, Ten Eyck PP, Meyerholz DK, Perlman S (2017). Sex-based differences in susceptibility to severe acute respiratory syndrome coronavirus infection. J Immunol.

[14] World Health Organization (2020). Sex, Gender and Influenza. https://apps.who.int/iris/bitstream/handle/10665/44401/9789241500111_eng.pdf?sequence=1&isAllowed=y.

[15] Wei X, Xiao Y-T, Wang J (2020). Sex differences in severity and mortality among patients with COVID- 19: evidence from pooled literature analysis and insights from integrated bioinformatic analysis [PREPRINT]. arXiv.

[16] Stokes EK, Zambrano LD, Anderson KN (2020). Coronavirus disease 2019 case surveillance—United States, January 22-May 30. MMWR Morb Mortal Wkly Rep.

[17] (2020). COVID-19: emerging gender data and why it matters. https://data.unwomen.org/resources/covid-19-emerging-gender-data-and-why-it-matters.

[18] Klein SL, Flanagan KL (2016). Sex differences in immune responses. Nat Rev Immunol.

[19] Schurz H, Salie M, Tromp G, Hoal EG, Kinnear CJ, Möller M (2019). The X chromosome and sex-specific effects in infectious disease susceptibility. Hum Genomics.

[20] Patel SK, Velkoska E, Burrell LM (2013). Emerging markers in cardiovascular disease: where does angiotensin‐converting enzyme 2 fit in?. Clin Exp Pharmacol Physiol.

[21] Chen L, Li X, Chen M, Feng Y, Xiong C (2020). The ACE2 expression in human heart indicates new potential mechanism of heart injury among patients infected with SARS-CoV-2. Cardiovasc Res.

[22] Sims AC, Baric RS, Yount B, Burkett SE, Collins PL, Pickles RJ (2005). Severe acute respiratory syndrome coronavirus infection of human ciliated airway epithelia: role of ciliated cells in viral spread in the conducting airways of the lungs. J Virol.

[23] Shastri A, Wheat J, Agrawal S (2020). Delayed clearance of SARS-CoV2 in male compared to female patients: high ACE2 expression in testes suggests possible existence of gender-specific viral reservoirs [PREPRINT]. medRxiv.

[24] Johnson HD, Sholcosky D, Gabello K, Ragni R, Ogonosky N (2003). Sex differences in public restroom handwashing behavior associated with visual behavior prompts. Percept Mot Skills.

[25] Bertakis KD, Azari R, Helms LJ, Callahan EJ, Robbins JA (2000). Gender differences in the utilization of health care services. J Fam Pract.

[26] Shaw AC, Goldstein DR, Montgomery RR (2013). Age-dependent dysregulation of innate immunity. Nat Rev Immunol.

[27] Smits SL, de Lang A, van den Brand JM (2010). Exacerbated innate host response to SARS-CoV in aged non-human primates. PLoS Pathog.

[28] Li M, Li L, Zhang Y, Wang X (2020). An investigation of the expression of 2019 novel coronavirus cell receptor gene ACE2 in a wide variety of human tissues [PREPRINT]. Research Square.

[29] Sharma G, Goodwin J (2006). Effect of aging on respiratory system physiology and immunology. Clin Interv Aging.

